# Replicating measurements of total hemoglobin mass (tHb‐mass) within a single day: precision of measurement; feasibility and safety of using oxygen to expedite carbon monoxide clearance

**DOI:** 10.14814/phy2.13829

**Published:** 2018-09-11

**Authors:** James O. M. Plumb, Shriya Kumar, James Otto, Walter Schmidt, Toby Richards, Hugh E. Montgomery, Mike P. W. Grocott

**Affiliations:** ^1^ Respiratory and Critical Care Research Area NIHR Biomedical Research Centre University Hospital Southampton NHS Foundation Trust University of Southampton Southampton United Kingdom; ^2^ Centre for Human Integrative Physiology Faculty of Medicine University of Southampton Southampton United Kingdom; ^3^ Anaesthesia and Critical Care Research Unit University Hospital Southampton NHSFT Southampton United Kingdom; ^4^ Shackleton Department of Anaesthesia University Hospital Southampton NHSFT Southampton United Kingdom; ^5^ University of Southampton Medical School Southampton United Kingdom; ^6^ Division of Surgery and Interventional Science University College London London United Kingdom; ^7^ Department of Sports Medicine/Sports Physiology University of Bayreuth Bayreuth Germany; ^8^ Centre for Human Health and Performance Institute of Sport, Exercise and Health University College London NIHR University College London Hospitals Biomedical Research Centre London United Kingdom; ^9^ Department of Anesthesiology Duke University School of Medicine Durham North Carolina

**Keywords:** Blood volume, optimized carbon monoxide re‐breathing, plasma volume, red cell volume, total hemoglobin mass (tHb‐mass)

## Abstract

Hemoglobin concentration ([Hb]) is a function of total hemoglobin mass (tHb‐mass) and plasma volume. [Hb] may fall by dilution due to plasma volume expansion and changes in the perioperative period may therefore correlate poorly with blood loss. A simple, reliable, repeatable way to measure plasma volume and tHb‐mass would have substantial clinical utility. The “optimized carbon monoxide re‐breathing method” (oCOR) meets these criteria. However, it is recommended that a minimum of 12 h (when breathing room air) is left between repeat measurements. Twenty‐four subjects underwent 3 days of testing. Two oCOR tests were performed (T1 and T2), 3 h apart, with a different CO clearance method employed between tests aiming to keep the carboxyhemoglobin level below 10%. The primary aim was to ascertain whether tHb‐mass testing could be safely repeated within 3 h if carboxyhemoglobin levels were actively reduced by breathing supplemental oxygen (*PROC*_*A*_). Secondary aims were to compare two other clearance methods; moderate exercise (*PROC*_*B*_), or a combination of the two (*PROC*_*C*_). Finally, the reliability of the oCOR method was assessed. Mean (SD) tHb‐mass was 807.9 ± (189.7 g) (for T1 on day 1). *PROC*_*A*_ lowered the carboxyhemoglobin level from the end of T1 (mean 6.64%) to the start of T2 (mean 2.95%) by a mean absolute value of 3.69%. For *PROC*_*B*_ and *PROC*_*C*_ the mean absolute decreases in carboxyhemoglobin were 4.00% and 4.31%, respectively. The fall in carboxyhemoglobin between T1 and T2 was greatest in *PROC*_*C*_ *;* this was statistically significantly lower than that of *PROC*_*A*_ (*P *=* *0.0039) and *PROC*_*B*_ (*P *=* *0.0289). The test‐retest reliability for the measurement of total hemoglobin mass was good with a mean typical error (TE) of 2.0%. The oCOR method is safe and can be repeated within 3 h when carbon monoxide is suitably cleared between tests. Using oxygen therapy alone adequately achieves this.

## Introduction

Humans are obligate aerobes, the oxygen necessary for metabolism being carried by circulating hemoglobin, whose concentration [Hb] has traditionally, been used as a measure of the blood's oxygen carrying capacity.

In 1968, the World Health Organization (WHO) defined anemia as a [Hb] <130 g L^−1^ for men and <120 g L^−1^ for women (WHO scientific group, 1968). Despite evidence that these levels may actually be inappropriately low they are still widely accepted within clinical practice (Beutler and Waalen 2006; Zavorsky et al. 2012). Perioperative anemia is common and is associated with greater post‐operative morbidity and mortality (Musallam et al. 2011). This may in part be due to impaired oxygen delivery. Measures of preoperative cardiopulmonary fitness, measured using cardiopulmonary exercise testing (CPET) such as peak oxygen consumption (VO_2_peak) and oxygen consumption at the anaerobic threshold (VO_2_AT) are inversely related to postoperative outcome. Both VO_2_peak and VO_2_AT fall when tHb‐mass is reduced by venesection (Dellweg et al. 2008) and improve with red cell transfusion (Wright et al. 2014). However, hemoglobin concentration [Hb] is a function of the total mass of circulating hemoglobin (tHb‐mass) and the volume of plasma within which it is carried (PV) and, as such, tHb‐mass might be better correlated with patients’ CPET performance than [Hb]. In keeping with this, we have shown that [Hb] correlates modestly (Otto et al. 2013) or not at all (Otto et al. 2017a) with peak oxygen consumption (VO_2_peak) or oxygen consumption at the anaerobic threshold (VO_2_AT).

In disease states in which PV expansion is common such as chronic heart or liver failure, [Hb] correlates poorly with tHb‐mass (Otto et al. 2017b). tHb‐mass is also more stable than [Hb] over time, being unaffected by shifts in PV (which may be exaggerated when intravenous fluids are administered perioperatively) (Eastwood et al. 2008; Garvican et al. 2010a). Thus, in the perioperative setting changes in tHb‐mass may be a better guide to true blood loss than changes in [Hb]. There is an increasing recognition of the unsuitability of using [Hb] as a reliable guide to perioperative transfusion (Shander and Ferraris 2017; Plumb et al. 2016).

tHb‐mass can be measured directly using the so‐called optimized carbon monoxide (CO) re‐breathing method (oCOR) (see Methods and Appendix 1 for further details). In use since 2005, it has predominantly been applied to athletes in order to monitor responses to altitude training (Gore et al. 2013), but more recently has been used in clinical medicine (Otto et al. 2017a,b; Koponen et al. 2013; Wrobel et al. 2016). There is no “normal range” for tHb‐mass described for healthy subjects. Endurance athletes would hope to have upwards of 13 g kg^−1^ (males) and 11 g kg^−1^ (females).

In assessing the changes in PV and tHb‐mass over the perioperative period, it would be useful to make repeated measurements over relatively short timescales. However, to date, most authors recommend that the oCOR can only be repeated after a 12‐h interval (when breathing room air) (Schmidt and Prommer 2005), due to the concern that physiological clearance of administered carbon monoxide for the purposes of tHb‐mass measurement would, under normal conditions, be expected to take approximately 9 h (based on a post test COHb level of 7% and a half‐life of 4.8 h) (Zavorsky et al. 2012). To address this, various methods have been proposed which might enhance carbon monoxide clearance rates (Zavorsky et al. 2012; Naef et al. 2015) such that tHb‐mass assessment could be more rapidly repeated while still maintaining carboxyhemoglobin levels below the proposed recommended maximum value of 10% (Otto et al. 2017a,b; Naef et al. 2015; Turner et al. 2014). With carbon monoxide toxicity exposure, concentration and time need to be considered; higher levels for a very short duration are less dangerous than lower levels for prolonged periods. In this regard, mild exercise (inducing a higher rate of pulmonary ventilation) and normobaric “hyperoxia” (supplementing oxygen at FiO_2 _> 0.21) are two potentially effective candidate interventions to facilitate the clearance of serum carboxyhemoglobin (Prommer et al. 2008). An important study by Zavorsky et al. (2012) demonstrated that mild exercise, hyperoxia, and increased pulmonary ventilation the so called “triple therapy” was most effective at reducing the half‐life of CO administered to healthy subjects. They also found that moderate exercise (in room air) was as effective as breathing 100% oxygen at rest at clearing CO. To the best of our knowledge, only one study (in healthy athletic males), using administration of supplemental inhaled oxygen during physical activity, has demonstrated the feasibility of doing this in the context of tHb‐mass assessment – here the efficacy of oxygen therapy alone, or of exercise alone was not assessed (Naef et al. 2015).

The primary aim of this study was to ascertain whether tHb‐mass testing could be safely repeated within 3 h if carboxyhemoglobin levels were actively reduced by breathing supplemental oxygen alone (*PROC*_*A*_). The secondary aims were to compare two other carboxyhemoglobin clearance procedures (gentle cycling alone, or gentle cycling in combination with oxygen administration‐procedure B and C (*PROC*_*B*_ and *PROC*_*C*_), respectively. The final aim was to evaluate the reliability of duplicate tHb‐mass measurements in healthy volunteers, who were not the elite or “sub elite” athletes largely studied by others previously (Naef et al. 2015).

## Methods

The study took place at University Hospital Southampton NHS Foundation Trust between August 2016 and March 2017.

Ethical approval was granted by the West Midlands (Edgbaston) Research Ethics Committee and NHS Health Research Authority (REC reference: 16/WM/0274). Local permissions were received from the University of Southampton (ERGO ID: 19642), University Hospital Southampton NHS Foundation Trust (R&D CRI 0329) and Southampton Centre for Biomedical Research Clinical Research Facility. The study was performed in accordance with the ethical standard set by the Declaration of Helsinki. Written informed consent was obtained from all participants.

Healthy adults aged over 16 years who were physically able to perform the testing protocol were eligible for recruitment. Excluded were adults lacking mental capacity to consent, pregnant women, smokers, prisoners, subjects with a baseline carboxyhemoglobin level >5%, or patients with hemoglobinopathies. One subject was a smoker (this was not clear until after the first experiments had taken place and their data were removed). No subjects had stayed at an altitude higher than 1500 m for any time in the preceding year. This left data from 24 subjects for analysis. In total, 24 x 6 (144) measurements of tHb‐mass were planned but 136 were carried out; the missing eight experiments were due to CO gas running out mid‐experiment in three subjects and a blood sample clotting in one subject. Of these 136 measurements, 122 were suitable for analysis. Values being discarded if the rise in carboxyhemoglobin (ΔCOHb%) was <4.5% (standard practice in our laboratory), if significant leaks were detected (CO ppm > 5 using the Dräger Pac 7000), if the breath‐holding technique was sub‐optimal (determined either by a leak using the Dräger Pac 7000 or if the subject failed to breath hold for 10 seconds), or if the resting carboxyhemoglobin values were greater than 5% (Appendix 3). Fourteen subjects had three consecutive days of measurement, the remaining 10 had all 3 days within a 10‐day period.

**Figure 1:**
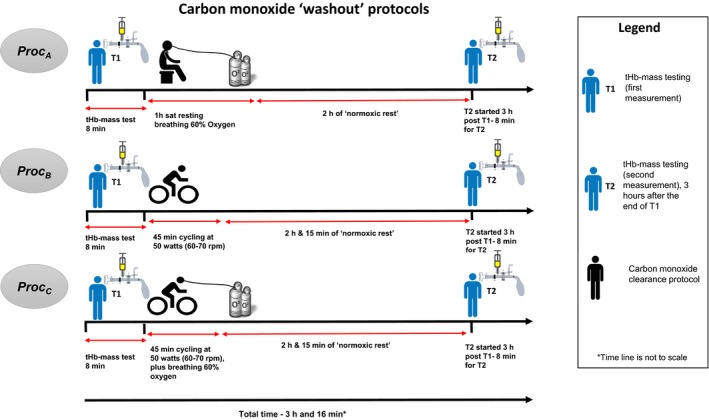
Schematic to describe the experimental sequence.

tHb‐mass was measured twice (a period of 3 h separating the first (T1) and second (T2) measurements) using the optimized carbon monoxide rebreathing (oCOR) method. This protocol was repeated on each of three separate occasions, with a different protocol to enhance CO clearance being applied between tests on each day. Figure 1 outlines the experimental design and study activity. The three experimental days had to occur within a 10‐day period.

### Day 1

Procedure A (*PROC*_*A*_) – Immediately after the first oCOR test, 60% oxygen was administered to seated subjects via a Venturi mask (Intersurgical Ltd, Berkshire, UK) for exactly 1 h. An identical oCOR test was repeated exactly 2 h later (3 h in total from the end of the first oCOR test).

### Day 2

Procedure B (*PROC*_*B*_) – Immediately after the first oCOR test, subjects exercised at low intensity (50 W and 60–70 revolutions per min) for 45 min on a static exercise cycle ergometer (Ergoline Ontibike 200, Germany). An identical oCOR test was repeated exactly 2 h and 15 min later (3 h in total from the end of the first oCOR test).

### Day 3

Procedure C (*PROC*_*C*_) – Immediately after the first oCOR test, subjects exercised as for *PROC*_*B*_ were positioned on a static exercise cycle ergometer (Ergoline Ontibike 200) while breathing 60% oxygen via a Venturi mask (Intersurgical Ltd, Berkshire). An identical oCOR test was repeated exactly 2 h and 15 min later (3 h in total from the end of the first oCOR test).

The fraction of inspired oxygen chosen was 0.6, this was for pragmatic and safety reasons. First, there is a convenient venturi delivery device that consistently delivers 0.6 available. Higher inspired fractions of oxygen can be detrimental if breathed for long periods and in a small subset of patients can be very detrimental if they are known to retain carbon dioxide (Ridler et al. 2014; Martin and Grocott 2013). We wanted to balance safety with expedient expulsion of CO gas.

In all cases, subjects were allowed to leave the lab between tests, remaining in the hospital and avoiding formal or strenuous physical activity. Finally, triplicate measurements of tHb‐mass taken on separate days (T1 of each procedure) were compared. We deliberately did not include a control procedure for clearance (i.e., no exercise and no inspired additional oxygen). It has previously been demonstrated that clearance of CO under these normal conditions is prolonged and the study by Naef et al. (2015) demonstrated this precisely.

### Optimized carbon monoxide rebreathing method

Subjects completed a baseline carbon monoxide rebreathing test (oCOR) (details of this method can be found in Appendix 2). Briefly, subjects were seated and inactive for 15 min prior to sampling. Subjects inhaled 0.8–1 mL kg^−1^ of CO mixed with 3 L of 100% oxygen via a glass spirometer (BloodTec, Bayreuth, Germany) and then rebreathed (via a CO_2_ scrubber) for 2 min while wearing a nose clip. A portable CO gas detector (Dräger Pac 7000, Drägerwerk AG & Co. KGaA, Germany) was used during the rebreathing period to check for possible CO leakage at the nose, mouthpiece, and spirometer.

Prior to commencing the rebreathing technique, the investigators inserted an intravenous cannula into the subject's upper limb. Venous blood samples were taken via Na‐heparinized syringes (RAPIDLyte, Siemens Healthcare Diagnostics Inc, USA) before (at baseline) and at 6 and 8 min after administration of CO gas. All three blood samples (0, 6, and 8 min), were analyzed using a laboratory blood gas analyzer (Radiometer, ABL800 FLEX) for carboxyhemoglobin percentage values. Each sample was analyzed three times within 1 h of collection. The analyzer used in this study was subjected to regular maintenance and quality control checks; the accuracy of which has been evaluated elsewhere (Turner et al. 2013). [Hb] and hematocrit values were measured using HemoCue (HemoCue AB, Radiometer, Sweden) and blood gas analyzer (Radiometer, ABL800 FLEX, Copenhagen), respectively.

The desired rise in carboxyhemoglobin is 5– 6.5% after administration of carbon monoxide gas aiming to achieve levels below 10% at the end of the test. This represents a tradeoff between precision and safety. However, “safe levels” of COHb are not exclusively about peak COHb% but duration of high blood levels is equally, if not more important. Most hemoximeters estimate carboxyhemoglobin only to a single decimal place meaning that smaller changes in carboxyhemoglobin could result in lower precision of measurement. We use a minimum Δ%COHb of 4.5% to avoid overestimating tHb‐mass. The safety limit of 10% is based on previous work by our group and professor Schmidt who developed the oCOR test. This level allows a significant margin of safety before subjects are likely to experience any significant level of CO toxicity. Previous researchers have also used this level (Otto et al. 2017a,b; Turner et al. 2014; Naef et al. 2014). Levels greater than 5% can produce symptoms such as mild headaches and when levels start to exceed 15% most individuals start to experience headaches and visual evoked potential start to change (Stewart 1975).

### Statistical analysis

Statistical analysis was performed using GraphPad Prism (version 7.0c for Apple Macintosh OSX) and SPSS Statistics (version 25 for Apple Macintosh Chicago, IL). The Shapiro‐Wilk test for normal distribution was used. Values are presented as mean ± standard deviation (SD), unless otherwise stated. Median and interquartile range (IQR) are reported when variables were not normally distributed. Categorical variables are presented as frequency (%). Differences in carboxyhemoglobin were assessed using the Student's paired *t*‐test.

This was a feasibility study so a formal power calculation was not required. The aim was to recruit 25 subjects on the advice of professor Schmidt (personal communication) based on the work of their group having performed thousands of tHb‐mass measurements. They use a number of 10 test/retests to ensure personal operator quality assurance. The statistical principles of test/retest reliability measures as described by Hopkins (2000) would also suggest that 10 patients are sufficient to get a meaningful estimate of typical error (TE) for an individual tester.

Test‐retest data (repeated measures in one and the same patient) are presented using Bland–Altman plots with limits of agreement (Bland and Altman 1986). Additionally, a specific approach to compute reliability statistics to compare test‐retest performance was used see (Appendix 4 for further detail) (Hopkins 2000). TE of measurement for tHb‐mass was calculated and expressed as coefficient of variation with 95% confidence limits (CL), derived from χ^2^ distributions. All tests were two‐sided and statistical significance was set at *P *<* *0.05. TE includes random error (analytic error arising from using the method‐specific apparatus and intra‐individual biological variation) but not systematic error (bias) (Gore et al. 2013; Naef et al. 2015; Gore et al. 2005). Reported studies using the oCOR have commonly reported this method (Naef et al. 2015; Fagoni et al. 2018; Keiser et al. 2017; Garvican et al. 2010b; Eastwood et al. 2012).

## Results

### Baseline characteristics

Twenty‐four subjects were included in the study (20 male), with age (median [range]) 23 [20–32] years, height 176 ± 11 cm, and weight 78.4 ± 12.8 kg. On day one, test 1, the average results were; [Hb] 152.4 ± 13.6 g L^−1^, hematocrit (Hct) 46.47 ± 4.3%, and tHb‐mass 807.9 ± 189.7 g.

### Comparing the three carboxyhemoglobin clearance procedures (*PROC*_*C*_, *PROC*_*B*_, and *PROC*_*C*_)

Table 1 shows the changes in carboxyhemoglobin for each procedure and the reliability statistics. All three carbon monoxide clearance methods achieved a reduction in carboxyhemoglobin from the end of T1 to the beginning of T2 on each of the days studied.

Baseline carboxyhemoglobin levels were higher at the start of T2 than T1 for all three procedures (median 1.15% (start of T1) vs. 2.87 % (start of T2), respectively, *P *<* *0.0001). T1 baseline carboxyhemoglobin was lower in *PROC*_*A*_ than in *PROC*_*B*_ and *PROC*_*C*_ (*P *=* *0.0010 and 0.0013, respectively). T2 baseline carboxyhemoglobin was lowest in *PROC*_*C*_ and it differed significantly from *PROC*_*A*_ and *PROC*_*B*_ (*P *=* *0.0039 and 0.0289, respectively). There were no significant differences between *PROC*_*A*_ and *PROC*_*B*_.

**Figure 2:**
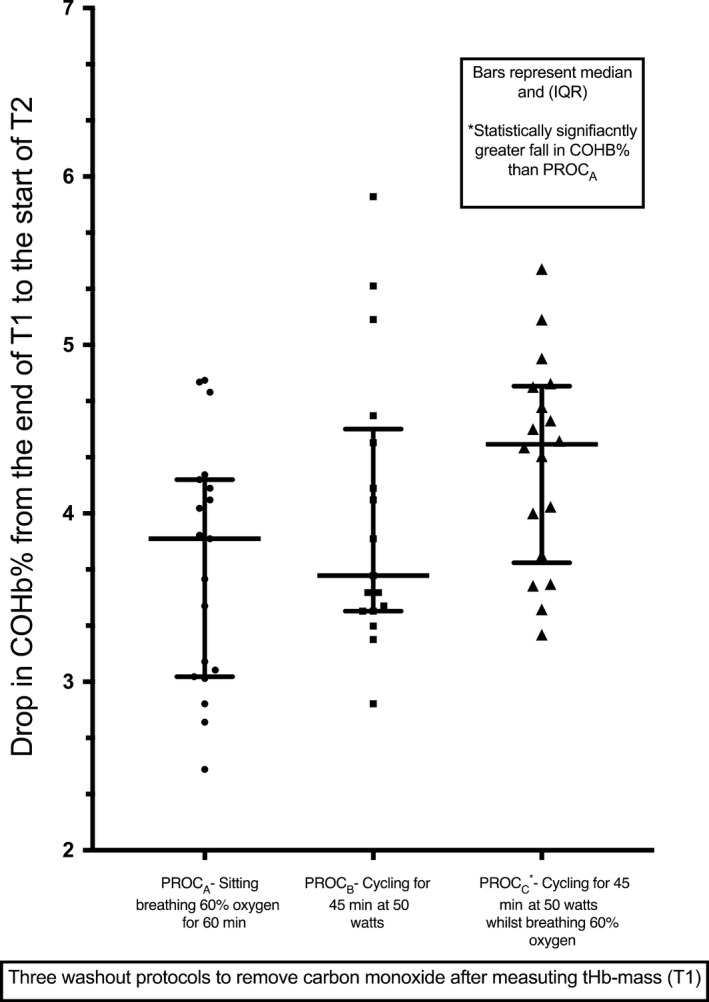
Comparing the fall in carboxyhemoglobin (COHb%) between test 1 and test 2 of each procedure (T1 and T2). Median with interquartile range shown for each procedure. *Denotes the significant difference between *PROC*_C_ and *PROC*_A_ (after multiple comparison correction using the Bonferroni method). COHb, carboxyhemoglobin; *PROC*_A/B/C_ procedures A/B/C.

The fall in carboxyhemoglobin between T1 and T2 (4.31%‐ absolute mean value for *PROC*_*C*_) was greater in *PROC*_*C*_ than *PROC*_*A*_ (absolute mean value 3.69%) (*P *=* *0.0039, (the difference between *PROC*_*C*_ and *PROC*_*A*_ being 0.62%) and this result was robust to analysis using one‐way ANOVA with Bonferroni multiple comparison test (*P = *0.037) (See Table 1). Although *PROC*_*B*_ and *PROC*_*C*_ were initially significantly different this was not the case after applying the Bonferroni test *P* = 0.617 (Fig. 2). The absolute mean fall between tests 1 and 2 for *PROC*_*B*_ was 4.00%. Cycling with supplemental oxygen (*PROC*_*C*_) was therefore statistically more efficient than oxygen alone (*PROC*_*A*_) but not statistically more efficient than cycling alone (*PROC*_*B*_) at lowering carbon monoxide levels in the blood. There was no statistical difference between oxygen alone (*PROC*_*A*_) and cycling alone (*PROC*_*B*_) at lowering carbon monoxide levels in the blood.

Differences between males and females revealed a mean ± SD tHb‐mass of 863.3 ± 147.0 and 541.3 ± 27.0, respectively. Further breakdown of male and female differences are tabulated in Table 2. All three procedures were relatively more efficient at clearing CO in females (see Table 2). Comparing *PROC*_*C*_ with *PROC*_*A*_ *and PROC*_*B*_ they differed statistically as above in males (*P *=* *0.0458 and *P* = 0.0072) but this did not hold true for females (*P *=* *0.8690). There were no other statistically significant differences between the procedures when males and females were separated.

### Test re‐test reliability

**Figure 3:**
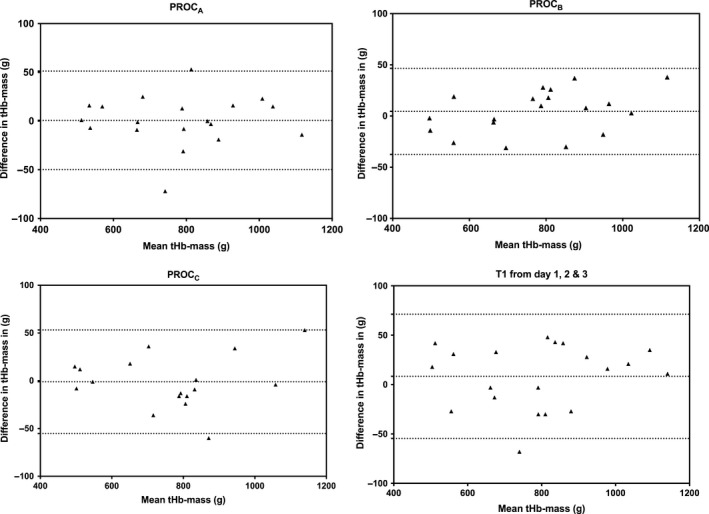
Bland‐Altman plots comparing tHb‐mass measurements between T1 and T2 for each procedure.

The overall TE for the study was 2.0%, 95% CI (1.67–2.59). Neither the procedure nor the baseline carboxyhemoglobin level influenced the calculated tHb‐mass. The TE of *PROC*_*A*_ *‐*_*C*_ are shown in Table 1 and Figure 3 shows the Bland–Altman plots for *PROC*_A–C_ (Bland and Altman 1986) with limits of agreement. The bias is similar for PROC_A‐C_ (0.7, 4.5, and −1.1, respectively), but increased when T1 was compared across the 3 days (8.4). There were no significant differences between subjects that had the measurements over three consecutive days (14 subjects) versus within 10 days (11 subjects) with regard to the precision of repeat measures of tHb‐mass.

**Figure 4:**
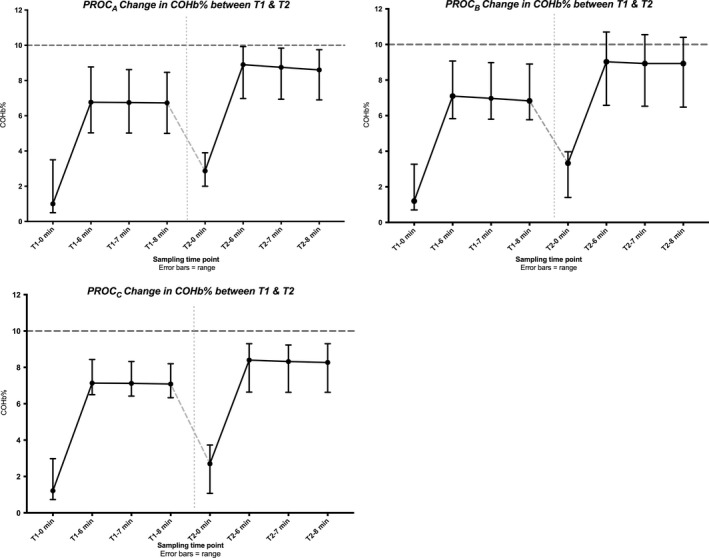
Change is serum carboxyhemoglobin (%) over test 1 and 2 (T1 and T2) of each procedure. Results are presented as median and range. Safety of duplicating the oCOR method is dependent on ensuring that the clearance procedure removes enough COHb after T1 to avoid excess COHb (>10%, red dotted line) during T2. Seven‐minute values are calculated and not measured values but are included as they are used to calculate COHb. COHb: carboxyhemoglobin; oCOR, optimized carbon monoxide rebreathing technique; *PROC*_A/B/C_, Procedure A/B/C, T1: test 1 or first measurement of the day, T2: test 2 or second measurement of the day.

Serum carboxyhemoglobin during *PROC*_*A*_ and *PROC*_*C*_ remained below 10% and ranged from 0.5 to 9.9% and 0.7 to 9.3%, respectively. Carboxyhemoglobin during *PROC*_*B*_ ranged from 0.7 to 10.7% (Fig. 4). One subject had a serum carboxyhemoglobin above 10% (10.7% at 6 min and 10.4% at 8 min) after carbon monoxide administration during T2.

**Table 1:** The three clearance protocols for washing out carbon monoxide after total hemoglobin mass measurement

	First measurement (T1)				Second measurement (T2)				Fall in COHb% from the end of T1 to the start of T2	Reliability statistic‐ Typical Error (TE) (95% CL)
Procedure	Baseline COHb (%)	COHb 7‐min value (%)	Delta change in COHb (%)	tHb‐mass (g)	Baseline COHb (%)	COHb 7‐min value (%)	Delta change in COHb (%)	tHb‐mass (g)		Overall 2.0 (1.67–2.59)
*Proc*_*A*_	1.09 ± 0.63	6.64 ± 0.90	5.55 ± 0.60	807.9 ± 189.7	2.95 ± 0.55	8.45 ± 0.83	5.50 ± 0.55	789.4 ± 171.2	3.69 ± 0.72	1.61 1.21–2.41
*Proc*_*B*_	1.39 ± 0.59	7.10 ± 0.80	5.67 ± 0.60	788.1 ± 180.1	3.10 ± 0.75	8.77 ± 0.96	5.67 ± 0.52	786.2 ± 172.5	4.00 ± 0.83	2.02 (1.53 –2.99
*Proc*_*C*_	1.33 ± 0.46	7.10 ± 0.43	5.75 ± 0.34	775.1 ± 188.5	2.72 ± 0.51	8.27 ± 0.72	5.63 ± 0.43	775.7 ± 181.2	4.31^a^^,^ b ^*,*^ c ± 0.61	2.37 (1.76 –3.6)

Mean carboxyhemoglobin (%) values at baseline and 7 min (the mean of min 6 and 8) during test 1 and 2 of each procedure. The Delta change from baseline to minute 7 and the T1–T2 fall in COHb% are shown. The resulting mean total hemoglobin mass is also shown. Typical error of measurement (TE) of tHb‐mass for the three carbon monoxide clearance procedures.

COHb, carboxyhemoglobin; *PROC*_A/B/C_, procedures A/B/C; TE, typical error of measurement; CL‐ confidence limits.

a The reason for slight discrepancy in the value generated by (COHb% 7‐min value from T1 – COHb% baseline value from T2) is that not all participants had values for both tests in each experiment. There were two participants who did not have a valid test in T1 but did have a value in baseline value in T2.

b Significantly different compared to the fall in COHb in *PROC*_A & B_ (*P* = 0.0039 and *P* = 0.0289, respectively).

c Statistically significant using one‐way ANOVA with Bonferroni multiple comparison test.

**Table 2:** Male and female differences between the three clearance procedures and resulting tHb‐mass

	Male	Female
Age (years)	Median 22, range 20–32	Median 22, range 22–23
Height (cm)	179.4 ± 8.9	161.0 ± 8.3
Weight (kg)	81.6 ± 11.4	63.0 ± 3.40
BMI (kg m^2^)	25 ± 3.4	24 ± 2.0
Hemoglobin concentration [Hb](g L^−1^)	154.5 ± 13.8	141 ± 2.60
tHb‐mass (g)	863.3 ± 147.0	541.3 ± 27.0
Blood volume (BV) (mL)	6188 ± 962.0	4220 ± 265.0
Plasma volume (PV) (mL)	3565 ± 664.3	2569 ± 182.2
Erythrocyte volume (EV) (mL)	2577 ± 524.0	1651 ± 83.20
Procedure A (*PROC*_A_) fall in COHb% from the end of T1 to the beginning of T2	3.54 ± 0.72	4.25 ± 0.38
Procedure B (*PROC*_B_) fall in COHb% from the end of T1 to the beginning of T2	3.90 ± 0.77	4.32 ± 1.10
Procedure C (*PROC*_C_) fall in COHb% from the end of T1 to the beginning of T2	4.20 ± 0.63	4.72 ± 0.34
*PROC*_A_ versus *PROC*_B_‐ statistically significant?	No *P* = 0.6543	No *P* = 0.8690
*PROC*_A_ versus *PROC*_*C*_‐ statistically significant?	Yes *P* = 0.0458	No *P* = 0.1119
*PROC*_B_ versus *PROC*_C_‐ statistically significant?	Yes *P* = 0.0072	No *P* = 0.5388

All values are expressed and mean ± standard deviation unless otherwise stated.

## Discussion

Repeated measurements of tHb‐mass can be safely made within 3 h if an adequate carbon monoxide clearance procedure is performed between tests. Administering oxygen alone (without exercise) is sufficient to achieve this and to avoid excess (>10%) carboxyhemoglobin levels being reached in healthy subjects.

Co‐administering oxygen with mild exercise lowered the carboxyhemoglobin the most (Fig. 2, Table 1 **)**. This was statistically different from oxygen alone but not from cycling alone (after application of Bonferroni's test). These data are in keeping with those from a study which evaluated the effectiveness of measures to treat carbon monoxide poisoning (Goldstein 2008) and of a study which showed “hyperoxic exercise” to be superior to 6 h of normal daily activity in reducing carboxyhemoglobin (Naef et al. 2015). They are also in keeping with the study by Zavorsky et al. (2012) which demonstrated that the “triple low” of exercise, supplemental oxygen, and increased pulmonary ventilation was the most effective way to clear CO from the blood. We found no statistical difference between *PROC*_*A*_ and *PROC*_*B*_ but did find that *PROC*_*C*_ was significantly better than *PROC*_*A*_ at lowering carboxyhemoglobin levels. The fact that there was no significant difference between the other procedures may have been a consequence of the study being underpowered to detect any real difference or may represent a lack of difference between these techniques.

When males and females were compared all three procedures lowered COHb% to a greater extent in female subjects. This is in keeping with previous research and the fact that females have a lower tHb‐mass. In a study examining this gender differences in CO t_1/2_ disappeared when tHb‐mass was normalized suggesting that CO storage explained much of the difference (Zavorsky et al. 2014). Due to the low number of female subjects within the study it is impossible to conclude further than this.

Strengths of this study include the fact that the same two operators performed all of the experiments in the same laboratory with the same equipment. Weaknesses include the fact that one subject performed the carbon monoxide clearance procedures in a different order. A relatively high number of experiments that were not completed (14/136) for diverse reasons (See Appendix 3). These findings do highlight that the oCOR technique is susceptible to technical difficulties, even when performed by experienced well‐trained technicians. It is possible that further refinement and the development of an automated oCOR technique in the future could improve this. Fourteen subjects were studied on consecutive days, and 10 over a period of up to 10 days. This is unlikely to have been of significance, given that tHb‐mass has been shown to be stable over days to months in contrast to hemoglobin concentration and hematocrit which are not (Eastwood et al. 2008; Garvican et al. 2010a). We did not measure minute ventilation (MV) after CO inhalation and are aware that this does limit our findings due to variances in MV affecting CO removal, with ventilation playing a significant role (Zavorsky et al. 2012). Another weakness was the period of rest post each procedure for additional washout. Pragmatically this was performed for safety reasons due to the concern that the baseline levels might be too high to test immediately post the washout procedure.

One subject had a carboxyhemoglobin level exceeding the accepted safety threshold of 10% Figure 4 (*PROC*_*B*_). They had no symptoms, and the carboxyhemoglobin level 1 hour later was safely below 10%. It should be noted that the safety limit used in this study (serum carboxyhemoglobin level of 10%) was a recommendation (Turner et al. 2014; Stewart 1975) also followed by the earlier study conducted by *Naef* and colleagues (Naef et al. 2015). We have used this level in all of our previous work and if a subject has exceeded 10% have treated them with supplemental oxygen until the level has reduced to below 10%. In fact, serum carboxyhemoglobin can be raised to 18% in healthy individuals without symptoms of toxicity developing, however the levels are lower in patients with heart disease (as one example) (Stewart 1975). As such, increasing serum carboxyhemoglobin to even 15% may also be acceptable. As most subjects had a serum carboxyhemoglobin less than 10% during all procedures, it is reasonable to believe that each of the three clearance methods was safe to use in this population. Much of this safety data are based on historical experiments and animal work (Stewart 1975; Stewart et al. 1973; Peterson and Stewart 1970). Our group alone has now conducted multiple hundreds of oCOR tests in healthy volunteer and clinical subjects without COHb levels exceeding 11% and without any adverse events being reported (Otto et al. 2017a,b). We have tested smokers in some of our previous work but do not have extensive data on levels and symptoms associated with carboxyhemoglobin levels in excess of 10%. The question of whether a top level of 10% is indeed to limit of “safety” for the oCOR method in healthy volunteers or clinical subjects was not focus of this study however we recognize that further controlled experiments examining this would be of use. This becomes relevant if multiple testing occurs within short time periods. Further work would be required to examine if the threshold of 10% could be safely exceeded.

### Reliability of duplicate total hemoglobin mass measurements

The overall typical error of repeat measurements within the same subject in this study of 2.0% (Table 2) is in keeping with the only published meta‐analysis on the subject, which reported a value of ~2.2% (Gore et al. 2013). This meta‐analysis is important as it groups together a number of small studies from different laboratories and totals 328 participants. If an individual has a tHb‐mass of 1000 g, a TE of 2.0% equates to a 20 g difference between tests. As such, the study by Naef et al. had a TE of 1.4% that would equate to only a 14 g difference. Our results were not quite as precise (Naef et al. 2015). The median total hemoglobin mass over all six oCOR tests was lower in our study than in that of Naef et al. (2015) (797 g vs. 914 g, respectively, equating to 16 g Hb vs. 13 g Hb based on a TE of 2.0 vs. 1.4) in keeping with our inclusion of four females and a less well‐trained population. It is conceivable that part of the difference is also due to the technical precision of the operators and/or the accuracy of the equipment. We used a rise in carboxyhemoglobin (ΔCOHb%) of >4.5%, some authors argue that higher values are required to improve precision (Burge and Skinner 1995).

Bland–Altman plots (Fig. 3) suggest reasonable precision. The biases were low (0.7, 4.5 and −1.1 for *PROC*_*A‐C*_, respectively) and higher when the first test of each day was compared (8.4).

### The future of the optimized carbon monoxide rebreathing method in anesthesia and perioperative care

The most promising discovery was that clearing carboxyhemoglobin through administration of oxygen alone was effective enough to ensure safe carboxyhemoglobin levels in nontrained individuals and to allow repeat testing within 3 h. This clearance method is likely to be the only option for many patients, whose co‐morbidities might preclude them from performing exercise. This may allow clinically useful serial measurements to assess blood volume, plasma volume, and tHb‐mass over the perioperative period, and thus the accurate quantification of blood loss. This extends the potential application of tHb‐mass testing in the clinical environment. In the future it may be possible to assess rapid changes in plasma or blood volume and tHb‐mass, such as might occur with surgery.

Future studies should seek to investigate the feasibility of duplicate tHb‐mass testing in a single day in specific patient groups, and in the perioperative period in particular. Modification of the oCOR for use in ventilated subjects in the theatre or intensive care environment would also be of great value.

This study represents a step toward successfully integrating the use of the oCOR method as a point‐of‐care clinical technique. It has become increasingly apparent that [Hb] (and thus the definition of anemia) is strongly influenced by plasma volume, which can be inappropriately expanded in certain disease states (Otto et al. 2017b). Therefore, efforts to readily measure tHb‐mass, as a means of deriving plasma content, are required in clinical medicine. The oCOR method is portable, minimally invasive, quick, and user‐friendly.

## Conclusion

The oCOR method is a safe technique that can be repeated within 3 h when carbon monoxide is suitably cleared between tests in healthy nonathletic individuals. Using oxygen therapy alone adequately achieves this. This method has minimal bias and good precision making it attractive for regular monitoring of blood volume derivatives. Clearance using oxygen alone can safely be used in healthy volunteers with a high degree of precision and safety and it is possible that this could translate to clinical subjects.

## Conflict of Interests

JP has received financial support from Siemens Healthcare Limited for consumables and hardware for research into the measurement of hemoglobin mass (2015‐2018). JP was given consumables from Intersurgical UK Ltd (2015‐2018); has received honoraria for speaking and/or travel expenses from Siemens and Vifor Pharma and has received unrestricted grant funding from Pharmacosmos. JP is unaware of any direct or indirect conflict of interest with the contents of this paper or its related fields. SK has no conflicts of interest. JO has no conflicts of interest. WS is a managing partner of the company “Blood tec GmbH,” but he is unaware of any direct or indirect conflict of interest with the contents of this paper. TR is director of the ironclinic.com. and has received research funding from a variety of sources, including; government, charity, and industry sources for research into anemia, blood transfusion, and iron therapy, including NIHR HTA, NHMRC, Health Foundation, Gideon Richter, Vifor Pharma Ltd, and Pharmaocosmos. He has also been an invited speaker at conferences and provided consultancy to government and industry on anemia, blood transfusion, and iron therapy in the last 5 year. Please see http://www.ucl.ac.uk for full list of disclosures. HM consults for Google Deepmind on health technology and is on the Council of the UK Intensive Care Society but is unaware of any direct or indirect conflict of interest with the contents of this paper or its related fields. MG is vice‐president of CPX International. He also serves on the medical advisor board of Sphere Medical Ltd and the board of EBPOM Community Interest Company, Medinspire Ltd and Oxygen Control Systems Ltd. He has received honoraria for speaking for and/or travel expenses from BOC Medical (Linde Group), Edwards Lifesciences and Cortex GmBH and unrestricted research support from Sphere Medical Ltd and Pharmacosmos Ltd. He leads the Fit‐4‐Surgery research collaboration and the Xtreme Everest oxygen research consortium, which has received unrestricted research grant funding from BOC Medical (Linde Group), Deltex Medical and Smiths Medical. MPWG was funded in part from the British Oxygen Company Chair of the Royal College of Anaesthetists awarded by the National Institute of Academic Anaesthesia. All funding was unrestricted. The funders had no role in study design, data collection, and analysis, decision to publish or the preparation of the manuscript. This work was conducted at the Southampton NIHR Biomedical Research Centre with subjects studied within the Southampton NIHR Clinical Research Facility.

## Appendix 1 Calculation of tHb‐Mass

tHb‐mass was calculated using a specifically design excel spreadsheet (Microsoft Excel 2011 for Apple Macintosh) using the formula:

1. tHb‐mass (g) = K × MCO(mL) × 100 × (Δ%COHb × 1.39)−1
2. K = barometric pressure × 760−1 × [1(0.003661 × temperature)]
3. MCO = COadm − (COsystem + lung (after disconnection) + COexhaled (after disconnection)
4. COadm = CO volume administered into the system
5. COsystem + lung (after disconnection) = CO concentration in spirometer × (spirometer volume + remaining volume in the lung after disconnection)
6. COexhaled (after disconnection) = end‐tidal CO concentration ×  alveolar ventilation × time
7. Δ%COHb = difference between baseline %COHb and %COHb post CO administration
8. (average of 6‐ and 8‐min %COHb values)
9. 1.39 = Hufner's number (constant) (mL CO×g Hb‐1)

Residual volume and alveolar ventilation were calculated according to “Standardized lung function testing. Official statement of the European Respiratory Society” (Standardized lung function testing, 1993). CO concentration is in parts per million (ppm).

Blood volume (BV), plasma volume (PV) and red cell volume (RCV) were calculated from mean corpuscular hemoglobin concentration (MCHC), [Hb] and tHb‐mass, as below:

1. BV (mL) = tHb‐mass (g)/[Hb] (g dL^−1^) × 100
2. RCV (mL) = tHb‐mass (g)/MCHC × 100
3. PV (mL) = BV−RCV

## Appendix 2 Measurement of tHb‐Mass Using the Optimized Carbon Monoxide Re‐Breathing Method (oCOR)

The use of CO to determine tHb‐mass was first proposed in the late 1800s, with refined techniques being published 100 years later (Thomsen et al. 1991). In 2005, Schmidt and Prommer reported a simpler and faster technique (described in detail below), which also required less blood sampling (Schmidt and Prommer 2005). It was applied almost exclusively, however, in the fields of athletic physiology, and thus failed to come to the attention of the bulk of the broader clinical/medical community.

tHb‐mass was determined using the validated oCOR method described in detail by Schmidt and Prommer (Schmidt and Prommer 2005). COHb concentration in blood was measured before and after 2 min rebreathing a known CO volume (0.5 to 1.0 mL kg^−1^ in this study depending on sex and [Hb]). Each participant was seated for 15 min to allow stabilization of plasma volume (PV), after which a mouthpiece connected them *via* a container of ‘soda lime’~10 g (carbon dioxide scrubber) to a spirometer (Spico‐CO Respirations‐Applicator, Blood Tec, Germany) and a 3‐L anesthetic bag pre‐filled with 100% oxygen. The patient exhaled to residual volume, breathed in the CO dose *via* the spirometer, held their breath for 10s, then continued normal breathing into the closed circuit *via* the spirometer for 1 min 50 sec. The participant then exhaled to residual volume, this exhaled volume being collected and analyzed to quantify the CO not absorbed into the bloodstream. Disconnected from the mouthpiece, participants finally fully exhaled to residual volume into a CO gas analyzer (Dräger Pac 7000, Drägerwerk AG & Co. KGaA, Germany) before and at 4 min after CO rebreathing, in order to determine the end tidal CO concentration and thereby the amount of CO exhaled after disconnecting the patient from the spirometer, that will also have not been absorbed into the blood.

## Appendix 3

A total of 144 experiments were planned. Only 136 took place, in seven experiments the test was not performed due to the CO gas supply running out and in one experiment the patient's blood clotted prior to analysis. Of the 136 experiments performed on 24 subjects, 122 were suitable to be included on the final analysis. Six experiments had a ΔCOHb% of <4.5% so they were discarded as per standard laboratory practice in our laboratory. Five experiments had a significant leak identified or gas was lost on injection (one experiment) and three experiments had technical problems with the blood gas analyzer calibration and were unsuitable due to inaccurate readings.

## Appendix 4

The Hopkins method for typical error calculation is described below. “A statistic that captures the notion of random variability in a single individual's values on repeated testing is the standard deviation of the individual's values. This within‐subject standard deviation is also known as the standard error of measurement. In plain language, it represents the typical error in a measurement.” (Hopkins 2000)

Following this approach, the test‐retest typical error is computed as follows:

1. Computed difference in total hemoglobin mass assessed in repeated tests.
2. Computed standard deviation of the difference in total hemoglobin mass assessed during the two tests.
3. Mean standard deviation divided by the square root of 2.

This is the method used by Professor Schmidt's group (pers. comm., 2018). A test‐retest typical error of around 2% is acceptable in the difference in tHb‐mass between repeat tests as described in the tables available at http://www.sportsci.org, written by Hopkins (2000). It should be noted that the confidence limits for the typical error are derived from a chi‐squared distribution. For small degrees of freedom, the upper limit tends to be skewed out relative to the lower limit. Tate and Klett provided an adjustment that reduces the skewness by minimizing the width of the confidence interval, although it is then not an equal‐probability interval. With only slight adjustment the Tate & Klett limits can be represented conveniently by a single factor (see Table II in Tate and Klett (1959)).
